# Better null models for assessing predictive accuracy of disease models

**DOI:** 10.1371/journal.pone.0285215

**Published:** 2023-05-05

**Authors:** Alexander C. Keyel, A. Marm Kilpatrick

**Affiliations:** 1 Division of Infectious Diseases, Wadsworth Center, New York State Department of Health, Albany, NY, United States of America; 2 Department of Atmospheric and Environmental Sciences, University at Albany, SUNY, Albany, NY, United States of America; 3 Department of Ecology and Evolutionary Biology, University of California, Santa Cruz, Santa Cruz, CA, United States of America; Gulf University for Science and Technology, KUWAIT

## Abstract

Null models provide a critical baseline for the evaluation of predictive disease models. Many studies consider only the grand mean null model (i.e. R^2^) when evaluating the predictive ability of a model, which is insufficient to convey the predictive power of a model. We evaluated ten null models for human cases of West Nile virus (WNV), a zoonotic mosquito-borne disease introduced to the United States in 1999. The Negative Binomial, Historical (i.e. using previous cases to predict future cases) and Always Absent null models were the strongest overall, and the majority of null models significantly outperformed the grand mean. The length of the training timeseries increased the performance of most null models in US counties where WNV cases were frequent, but improvements were similar for most null models, so relative scores remained unchanged. We argue that a combination of null models is needed to evaluate the forecasting performance of predictive models for infectious diseases and the grand mean is the lowest bar.

## Introduction

Forecasting infectious disease dynamics is a key challenge for the 21^st^ century [1]. Climate and land use change, combined with the introduction of pathogens to new regions, has created an urgent need for predicting future disease threats [2]. Large data sets and new modeling and statistical techniques have opened up possibilities for ecological forecasting [3]. A key step in the evaluation of predictive models is assessing their improvement over null models. The use of null models to provide a baseline in the absence of specific mechanisms has a long history in ecology [4]. Such baselines are important, as in some cases, predictive models may appear to be informative, but may be no better than a simple and uninformative null model [5, 6]. For example, when dealing with rare events, if a predictive model is outperformed by a null model that predicts the event to never occur, it is not providing much useful information about the process being studied [5].

West Nile Virus (WNV) is an excellent system in which to examine null models in a probabilistic context. WNV is a flavivirus that cycles between mosquito and avian populations [7–9]. WNV was introduced to the United States (US) in 1999 [10] and rapidly spread to the conterminous US and throughout the Americas [11]. As a nationally notifiable disease in the US, long-term data sets (>20 years) exist on human cases [12]. Many models have been built for predicting WNV risk [13] including mechanistic models based on climate and vector data sets [e.g., 14, 15]. Most studies of WNV, and many other pathogens, have included only a very simplistic null model (e.g. R^2^, which uses the grand mean of the training data) for assessment of model accuracy.

Our aim was to examine a range of null models (Table 1) to provide guidance on null model selection and performance in disease forecasting for locations with frequent (≥50% of years with disease) and infrequent cases (disease present, but <50% of years). We tested 10 null models using the number of WNV cases in each county in the US in each year in a probabilistic framework. Where cases were frequent and timeseries were long, we hypothesized the Negative Binomial model would perform the best due to its ability to model count distributions with a variable rate parameter. Where cases were infrequent and time series were short, we predicted that no models would significantly outperform the Always Absent mode.

**Table 1:** A short description of the ten null models examined.

**Always Absent Null** (non-probabilistic):	WNV is always predicted to be absent (i.e., the model always predicts 0 cases or incidence).
**Pooled Mean Value Null**^**1**^^,^^**2**^ (non-probabilistic):	WNV is predicted to have its mean value of cases.
**Mean Value Null** (non-probabilistic):	As in the Pooled Mean Value Null, but stratified by county.
**Prior Year Null** (non-probabilistic):	The results from the prior year are used to predict the current year [16].
**Historical Null**:	Prior observations for the location are sampled with replacement, creating a probabilistic distribution of future cases.
**Pooled Incidence Null**^**2**^:	The number of cases were predicted by sampling a binomial distribution, using incidence as the probability, and the county’s population as the size parameter. Incidence was calculated for the entire region of interest (i.e. the United States).
**Incidence Null**:	As in the Pooled Incidence Null, but incidence is calculated stratified by county, allowing the model to capture local hotspots of disease.
**Negative Binomial Null**^**3**^:	We estimated the mean and dispersion parameters of a negative binomial distribution using the human case counts in each year from each county Random draws for each county were then drawn using the estimated mean and dispersion parameter.
**Autoregressive AR1 Null**^**4**^:	An autoregressive model was fit. The model produced a mean estimate for the next time step and a normally-distributed error around that mean. We randomly sampled the normal distribution around the mean. All values less than zero were assigned to 0, as negative human cases are not possible.
**Uniform Null**:	Random draws were taken from a uniform distribution of cases bounded by 0 to the maximum number of cases observed in a county.

^1^ Model predictions would differ if a pooled mean incidence null were used. However, the Pooled Incidence model represents a more sophisticated version of that null model, hence we did not evaluate a non-probabilistic pooled mean incidence model.

^2^ Pooled models were fit with data from the entire study region. All other models were fit on a county-by-county basis.

^3^ Using the MASS package in R [17].

^4^ Using the arima function in R [18].

## Material and methods

### Data set

We compared the accuracy of 10 null models using the CDC Neuroinvasive WNV Case records (Source: ArboNET, Arboviral Diseases Branch, Centers for Disease Control and Prevention, Fort Collins, Colorado; contact the CDC for data access). This is a national data set of the number of WNV neuroinvasive disease cases in each county in each year from 3108 counties in the conterminous United States (US) from 2000–2021. We used WNV neuroinvasive cases, because there is less variability across different states in detection of these cases compared to WNV fever cases.

**Fig 1:**
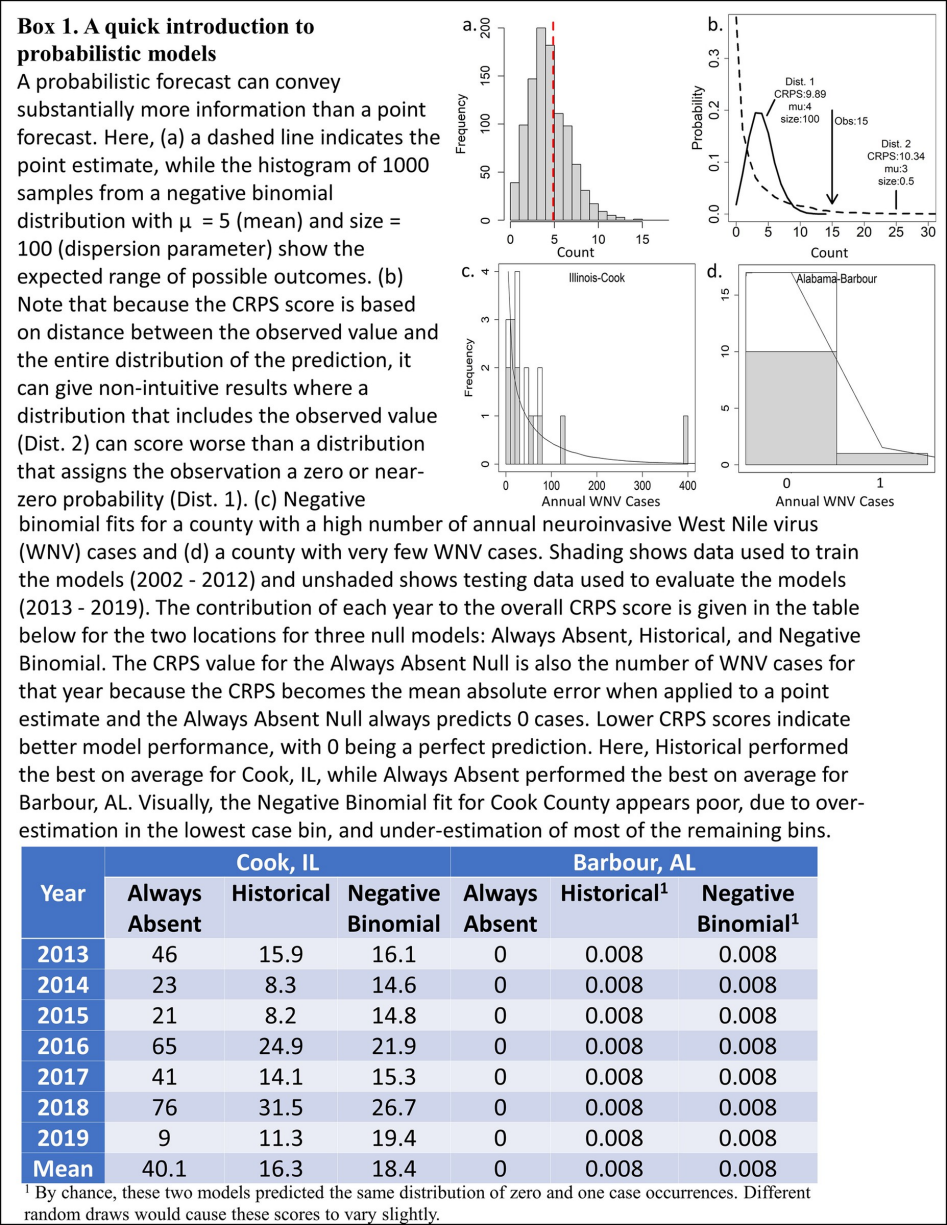
A quick introduction to probabilistic models.

We divided the data set into two groups: 159 counties that have had 11 or more years with WNV (frequent WNV set), and 1880 counties that had 1–10 years with WNV (infrequent WNV set). The 1069 counties that never had WNV cases were excluded to avoid zero-inflation. The first year a state reported a case of WNV (per [9]) was used as the first year of training data for all counties within that state. As a result, the number of counties included in the analysis increased over time (Table 2). Model predictions were made using at least 4 years of training data. We used the Continuous Ranked Probability Score, a probabilistic scoring approach that can evaluate a distribution of predicted outcomes (Fig 1). Population data for each year for the incidence-based null models came from the United States Census Bureau [19, 20]. Population data from 2019 were used for 2020 and 2021 as well, due to missing data for these years.

**Table 2:** Number of counties used for the null model analysis by prediction year for 2004–2021. After 2008, the sample size remained constant for all following years as all states had the minimum 4 years of training data with WNV by that point.

Year	N (frequent WNV)	N (infrequent WNV)
2004	25	213
2005	79	1042
2006	154	1831
2007	159	1865
2008–2021	159	1880

We also tested whether our model results were sensitive to the length of time series for selected models. Model years were selected at random (without replacement) to use as training data to predict a randomly selected focal year. This allowed us to disentangle length of time series from the specific order of observation of results. However, models that required a temporal structure were excluded from this analysis (i.e. Prior Year and Autoregressive). The Incidence and Pooled Incidence models were also excluded from this analysis as they used the prior-year’s population for converting incidence to case counts. Only data from 2005 and later were used in this analysis to ensure that WNV had already been established in all counties.

### Null models

The ten null models are described in Table 1. Note that using case counts versus incidence does not make a large difference to the outcome when stratifying by county for the mean value model, incidence model, prior-year model, or historical null model, because population is relatively consistent from one year to another, and therefore counts and incidence can be interconverted by multiplying or dividing by population. However, the choice of incidence or case counts as the model basis does lead to different outcomes when pooling across counties with different population sizes, or when working with count-based models such as the negative binomial.

### Scoring method

We used the Continuous Ranked Probability Score (CRPS), which is a proper scoring method [21, 22]. A proper scoring method is one that correctly assigns a better score to a better model in the long run. We chose the CRPS rather than the Logarithmic Score because the former scores forecasts based on the distance from each predicted probability to the observation, whereas the latter only scores whether an observation is within a bin or outside of a bin, with no consideration of how far outside the bin the prediction was [23–25]. As the CRPS scores are based on distance from the observed value, the models above were allowed to predict fractional cases of WNV (e.g. if the mean number of cases was 2.5 cases, that would be used as the prediction rather than rounding up or down to the nearest whole number). For null models that required sampling from a probabilistic distribution, we used 100 random draws. Data analyses were performed in R [26]. Code for running the null models is available via the probnulls package on GitHub (www.github.com/akeyel/probnulls/R/NullModels.R).

### Length of training time series

We examined how the length of the time series for training each null model affected its prediction using the mean of 10 CRPS scores of each of six null models (Always Absent, Pooled Mean Value, Mean Value, Historical, Uniform, and Negative Binomial) for each of 13 different training time series lengths (5 to 17 years). We regressed the mean CRPS score against the training time series length and included the null model as an additional predictor. We compared additive and interaction models of training time series length and null model by AIC. We performed the analysis separately for high and low incidence counties. We show the slopes and statistics for each null model using the *lstrends()* function from the *emmeans* package in R.

## Results

**Fig 2:**
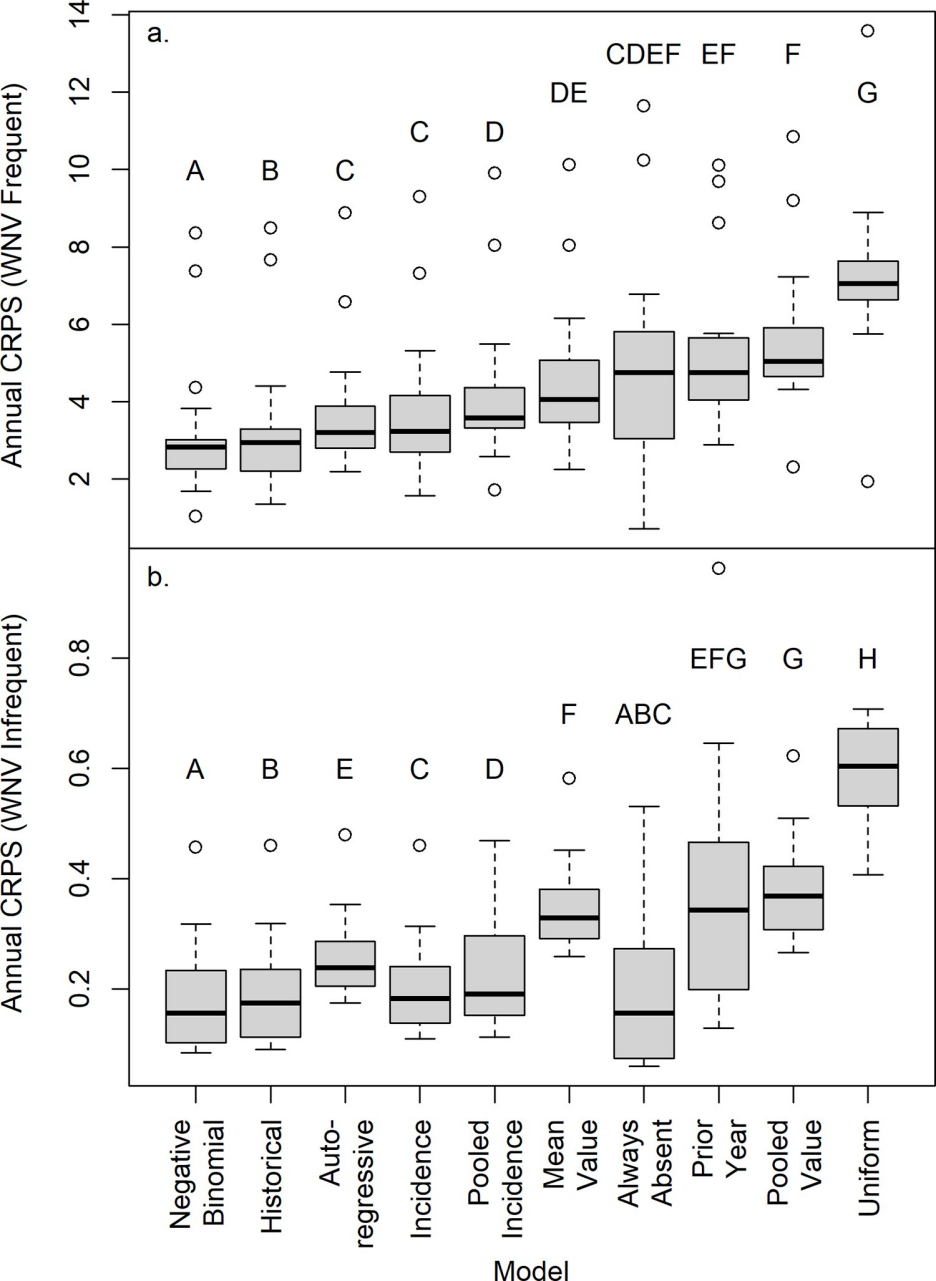
Continuous Ranked Probability Scores (CRPS) for 2004–2021 for 10 null models for a) counties with at least 50% of the time series with WNV (“Frequent”) and b) for counties with at least one case, but cases < 50% of the time series (“Infrequent”). The mean CRPS scores was calculated across all counties for each model and year, and the plot shows the median value of these mean annual CRPS scores by model, with the box showing the 25% and 75% quartiles, whiskers corresponding to +/- 1.5 times the Interquartile range, and circles corresponding to values outliers outside this range. Different letters indicate significant differences between models at α = 0.05 after a sequential Bonferroni correction for multiple comparisons [27].

The Mean Value null model (R^2^) that is frequently used as the baseline for prediction accuracy was among the weakest of the null models (Fig 2). It performed worse than 5 null models (significantly worse than 4; Fig 2) for frequent WNV counties and worse than 6 null models (significantly worse than 5) for infrequent WNV counties (Fig 2). In contrast, the Negative Binomial null model was significantly better than other null models for predicting neuroinvasive cases of WNV in the frequent WNV analysis (Fig 2A, paired t-tests using a Holm correction for multiple comparisons [27]). The Negative Binomial was also significantly better than eight other null models (all except the Always Absent model which was equally accurate) in the infrequent WNV analysis (Fig 2B). The Negative Binomial was the top model in 8 individual years (out of 18) for the frequent WNV analysis, and in 9 of 18 years for the infrequent WNV analysis (Table 3). The Historical Null model also performed very well in both frequent and infrequent WNV analyses across all years (Fig 2), and outperformed all other models in 5 individual years for frequent WNV counties (Table 3). Finally, in the infrequent WNV analysis, the Always Absent model was tied for the best model for all years combined, and outperformed seven other models (Fig 2B) and was the best model for 8 individual years (Table 3).

**Table 3:** Frequency of each model having the lowest CRPS score for a year in counties where WNV is frequent (>50% of time series) or infrequent (present < 50% of the time series).

Model	Frequent WNV	Infrequent WNV
Negative Binomial	8	9
Historical Null	5	0
Always Absent	2	8
Incidence	2	1
Autoregressive	1	0
Mean Value + 4 others^1^	0	0

^1^ Mean Value, Pooled Mean Value, Pooled Incidence, Uniform, and Prior Year null models were never the top model in either analysis

**Fig 3:**
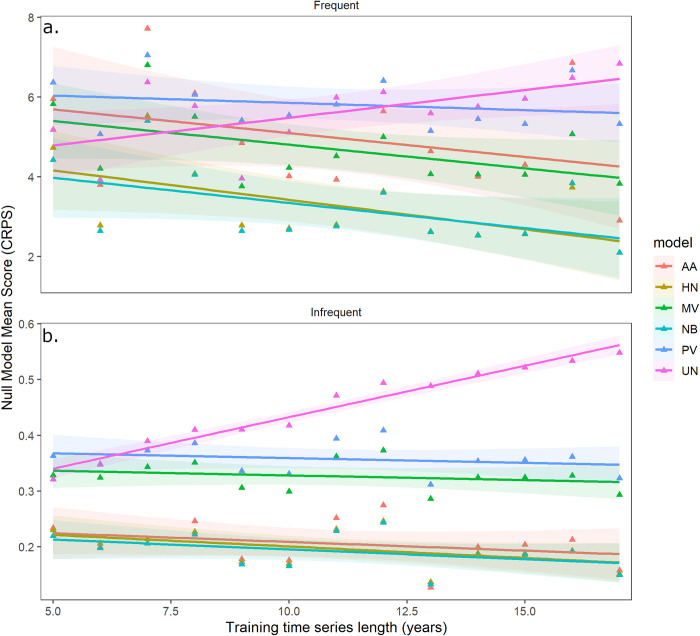
The Negative Binomial and Historical were generally the top two models (lower CRPS scores correspond to a more accurate model), independent of length of time series used to train the models for both a) the counties with frequent WNV and b) the counties with infrequent WNV cases. Training years were randomly selected from the entire time series, and a random focal year was selected for evaluation. Only a subset of null models was evaluated over time. Shading indicates a 95% confidence interval for the estimated mean. AA: Always Absent, HN: Historical Null, MV: Mean Value, NB: Negative Binomial, PV: Pooled Value, UN: Uniform.

The length of the training time series had only weak effects on null model performance (Fig 3). For frequent WNV counties, model score improved significantly with the length of the training time series for four of the six models examined, but the effect was similar for all four models (Table 4). For the two remaining models, increasing the length of the training time series had a non-significant improvement in model score (Pooled Mean Value) or actually made the score worse (Uniform) (Table 5). For infrequent WNV counties mean CRPS null model score did not improve significantly with the length of the training time series for any model, but got significantly worse for the Uniform Null (Table 5). Thus, except for the Uniform Null, the relative rankings of null models were the same across the full range of time series lengths examined (5 to 17 years; Fig 3).

**Table 4:** Analysis of the length of the training time series on the mean CRPS score for six null models for frequent WNV counties (Fig 3A). A model with an interaction between null model and time series length had more support than an additive model (ΔAIC = 21, see S1 Table in S1 File for detailed parameter estimates). The table shows the statistics for the slopes for each model (not differences between slopes).

Null Model	Estimate	SE	t value	P-value
Always Absent	-0.12	0.044	-2.74	0.0063
Historical	-0.15	0.044	-3.39	0.0007
Mean Value	-0.12	0.044	-2.73	0.007
Negative Binomial	-0.13	0.044	-2.90	0.0038
Pooled Mean Value	-0.0365	0.044	-0.84	0.40
Uniform	0.14	0.044	3.19	0.0015

**Table 5:** Analysis of the length of the training time series on the mean CRPS score for six null models for infrequent WNV counties (Fig 3B). A model with an interaction between null model and time series length had more support than an additive model (ΔAIC = 50, see S2 Table in S1 File for detailed parameter estimates). The table shows the statistics for the slopes for each model (not differences between slopes).

Null Model	Estimate	SE	t value	P-value
Always Absent	-0.0031	0.0025	-1.25	0.21
Historical	-0.0041	0.0025	-1.66	0.098
Mean Value	-0.0017	0.0025	-0.67	0.50
Negative Binomial	-0.0035	0.0025	-1.41	0.16
Pooled Mean Value	-0.0017	0.0025	-0.69	0.49
Uniform	0.018	0.0025	7.40	< .0001

## Discussion

At least five null models significantly outperformed a county-based grand mean and many did far better (Figs 2, 3). A grand mean calculated across all included counties (Pooled Mean model) performed even worse. Thus, when evaluating the performance of new statistical or mechanistic models of disease incidence, there are far better null models than the grand mean (i.e. R^2^). These null models can be easily calculated for time-series data (e.g., using the probnulls package from GitHub in R), and our results suggest that the length of time series was not critical for developing a robust null model across a range of 4–16 years. The Negative Binomial and Historical nulls were the strongest null models overall (Fig 2), with the Always Absent null performing well where disease cases were infrequent. The strong performance of the Always Absent null in regions where WNV was infrequent (statistically tied with Negative Binomial, Fig 2; top model in 8 of 18 years, Table 3) is a reminder that basic accuracy statistics for rare events can appear high.

The structure and scale of the underlying data may affect the performance of the different null models. The WNV data set here does not have a clear temporal trend. A strong temporal trend would likely have changed which model performed the best. Specifically, null models that use the recent past to predict future cases (e.g. autoregressive models) would perform much better. Seasonal patterns, as examined in recent dengue forecasts [1], could also affect which null model performs best. Future work could explore the performance of different models under different magnitudes of temporal trend and stochastic variation. Many (34%) of counties in the US did not have a neuroinvasive case within the study period. For risk estimates for these counties, fitting models on groups of counties may be necessary [e.g., as in 28]. Additionally, county-annual scales may be more relevant to academic study than to vector control and public health responses [29]. Research on null model performance is needed at finer spatial and temporal scales.

Broadly, null models are seeing increased use in the infectious disease modeling literature. A uniform model and a SARIMA model were used to predict dengue cases as part of a forecasting challenge in Puerto Rico [1]. A random walk and a probabilistic prior-week model were used as null models for forecasting COVID-19 deaths [30], and a modification of a simple AR(1) model was found to perform well for predicting COVID-19 hospitalizations [31, 32].

## Conclusion

We strongly recommend the inclusion of multiple null models when testing predictive models of vector-borne diseases. A grand mean calculated from the training data set is an inadequate null model given the suite of probabilistic alternatives available. The Negative Binomial and Historical nulls performed especially well for WNV and simple autoregressive models performed moderately well and would likely perform even better for data with temporal trends. Negative Binomial and Historical null models performed well both when WNV cases were frequent and when they were infrequent, and their relative performance did not depend on the length of the training time series. Researchers proposing mechanistic models should determine if their models are an improvement over a simple statistical description of historical patterns.

## Supporting information

S1 File Two tables containing full parameter details for the time series length analysis for counties with frequent (S1 Table) and infrequent (S2 Table) WNV cases. (DOCX)
