# Supplementary material for: Better null models for assessing predictive accuracy of disease models
Source: PLoS One. 2023 May 5;18(5):e0285215. doi: 10.1371/journal.pone.0285215 (PMC10162537; doi:10.1371/journal.pone.0285215)
Supplement: S1 File — (DOCX) [file pone.0285215.s001.docx]

# **Supplemental Material for: Better null models for assessing predictive accuracy of disease models**

Authors: Alexander C. Keyel^1,2^ and A. Marm Kilpatrick^3^

^1^ Division of Infectious Diseases, Wadsworth Center, New York State Department of Health, Albany, NY, United States of America

^2^ Department of Atmospheric and Environmental Sciences, University at Albany, SUNY, Albany, NY, United States of America

^3^ Department of Ecology and Evolutionary Biology, University of California, Santa Cruz, CA

**Table S1.** Analysis of the length of the training time series on the CRPS score for six null models for frequent WNV counties (Figure 2a). A model with an interaction between null model and time series length had more support than an additive model (ΔAIC = 21).

| Predictor | Estimate | SE | t value | P-value |
| --- | --- | --- | --- | --- |
| Intercept (Neg Binomial) | 4.61 | 0.51 | 9.09 | < 2e-16 |
| Length of time series | -0.13 | 0.044 | -2.90 | 0.0038 |
| Always absent | 1.68 | 0.72 | 2.35 | 0.019 |
| Historical | 0.29 | 0.72 | 0.41 | 0.68 |
| Mean | 1.39 | 0.72 | 1.94 | 0.053 |
| Pooled mean | 1.61 | 0.72 | 2.25 | 0.025 |
| Uniform | -0.52 | 0.72 | -0.73 | 0.47 |
| Length*Always absent | 0.0070 | 0.062 | 0.11 | 0.91 |
| Length*Historical | -0.021 | 0.062 | -0.35 | 0.73 |
| Length*Mean | 0.0076 | 0.062 | 0.12 | 0.90 |
| Length*Pooled mean^1^ | 0.090 | 0.062 | 1.46 | 0.15 |
| Length*Uniform | 0.27 | 0.062 | 4.31 | 1.86E-05 |

^1^ If the analysis is releveled to the Pooled Mean model, the length of time series variable is not significant for this model (p = 0.40).

**Table S2.** Analysis of the length of the training time series on the mean CRPS score for six null models for infrequent WNV counties (Figure 2c). A model with an interaction between null model and time series length had more support than an additive model (ΔAIC = 50).

| Predictor | Estimate | SE | t value | P-value |
| --- | --- | --- | --- | --- |
| Intercept (Neg Binomial) | 0.23 | 0.029 | 7.95 | 6.54E-15 |
| Length time series (NB) | -0.0035 | 0.0025 | -1.41 | 0.16 |
| Always absent | 0.010 | 0.041 | 0.23 | 0.82 |
| Historical | 0.011 | 0.041 | 0.28 | 0.78 |
| Mean | 0.11 | 0.041 | 2.79 | 0.0055 |
| Pooled mean | 0.15 | 0.041 | 3.55 | 0.00041 |
| Uniform | 0.017 | 0.041 | 0.42 | 0.67 |
| Length*Always absent | 0.00039 | 0.0035 | 0.11 | 0.91 |
| Length*Historical | -0.00062 | 0.0035 | -0.17 | 0.86 |
| Length*Mean | 0.0018 | 0.0035 | 0.52 | 0.60 |
| Length*Pooled mean | 0.0018 | 0.0035 | 0.51 | 0.61 |
| Length*Uniform | 0.022 | 0.0035 | 6.23 | 7.77E-10 |
